# Participant food tracking in psychiatric ketogenic metabolic therapy: an unresolved clinical, psychosocial, and implementation question

**DOI:** 10.3389/fnut.2026.1879746

**Published:** 2026-08-06

**Authors:** Nicole Laurent

**Affiliations:** Independent Researcher, Vancouver, WA, United States

**Keywords:** conceptual analysis, dietary self-monitoring, food records, ketogenic diet, ketogenic dietary therapy, ketogenic metabolic therapy, metabolic psychiatry, serious mental illness

## Abstract

Participant food tracking in psychiatric ketogenic metabolic therapy (KMT) varies across the metabolic psychiatry literature but is reported inconsistently rather than examined as a delivery decision with its own clinical rationale and consequences. This conceptual analysis develops a framework for participant food tracking in psychiatric KMT using metabolic psychiatry literature to identify variation in delivery models and draws on adjacent literatures relevant to dietary recording and reporting guidance for complex nonpharmacologic interventions. The framework is organized around three unresolved clinical questions: what assumptions about patient capacity shape who is taught to track and why; whether tracking functions as burden and/or treatment work, with implications for treatment ownership and continuity; and how short-term professional management relates to long-term treatment independence. Research priorities and potential methodologies are organized across ten interrelated domains that follow from these questions. To situate these questions, a supporting continuum maps variation in participant involvement across delivery approaches, from professionally managed models with no participant-generated record to higher levels of participant involvement in dietary recording. The framework offers a shared way to examine how participant food tracking is understood, assigned, and used in psychiatric KMT, and to investigate the clinical and psychosocial functions it serves within treatment.

## 1 Introduction

Metabolic psychiatry is an emerging field focused on the intersection of metabolism and mental health that investigates bidirectional relationships between metabolic and psychiatric processes to inform new interventions for psychiatric disorders (1, 2). Ketogenic metabolic therapy (KMT), also known as the ketogenic diet (KD) or ketogenic dietary therapy (KDT), is a clinician supervised dietary intervention that induces and maintains nutritional ketosis through carbohydrate restriction and a higher fat dietary pattern, with structured education, monitoring, and safety management integrated into care (3, 4). KMT has expanded to adjunctive psychiatric care as a transdiagnostic metabolic intervention (2, 5), with published applications that include major depressive disorder, bipolar disorder, schizophrenia-spectrum conditions, post-traumatic stress disorder, obsessive-compulsive disorder, and related psychiatric presentations (6–11). As KMT research and clinical application moves into multiple diagnoses, settings, and delivery contexts, questions about how the intervention is implemented have become as clinically relevant as questions of treatment efficacy. Current KMT delivery models vary in the level of participant involvement in dietary implementation, and that variation may have consequences for burden, treatment fidelity, and the sustainability of treatment effects beyond periods of intensive clinical support.

Participant food tracking refers to participant-generated records of dietary intake. Across published psychiatric KMT studies, some participants receive fully provisioned meals and carry no independent tracking responsibility, while others are expected to monitor macronutrient intake per meal or daily use of food logging applications, weighed food records, or photo-based dietary journals. Between these approaches lies a range of intermediate models involving food lists, meal plans, exchanges, and heuristics that assign varying degrees of dietary implementation responsibility to the participant. This range is treated here as a descriptive continuum of participant involvement rather than as a binary distinction between tracking and no tracking. This variation may be clinically meaningful, but it has not yet been examined as such in the psychiatric KMT or the adjacent peer-reviewed literature in epilepsy. Participant food tracking is typically reported incidentally if at all and treated as a logistical detail rather than a delivery decision with its own clinical rationale and consequences.

Decisions about whether and how participants are taught to track may affect the cognitive and behavioral burden of treatment. The precision with which participants maintain dietary targets, and the degree to which they can sustain KMT independently after formal clinical support decreases or ends may have important implications that shape treatment persistence and post-discharge continuity in psychiatric populations. Food tracking, when taught as a skill beyond use to assess compliance, may also function as a form of behavioral activation, with dietary self-efficacy and treatment ownership as additional possible effects that predict continuation. Decisions about whether to teach tracking, including the level of precision and the degree of scaffolding involved, may therefore carry psychosocial consequences that extend well beyond macronutrient accuracy. Whether tracking functions as burden or as beneficial treatment work may also depend on the psychiatric presentation, the degree of functional impairment associated with psychiatric illness, and the degree to which treatment effects have begun to stabilize capacity and motivation. The optimal timing for introducing more precise food tracking instruction as treatment progresses remains unexplored in the psychiatric KMT literature and unaddressed in the epilepsy KDT implementation guidance reviewed here (12, 13). Nor has the role of support persons in initiating or scaffolding tracking on behalf of adults with psychiatric illness been evaluated. Whether such support facilitates or delays the acquisition of independent dietary self-management skills remains unknown. These implications remain insufficiently examined in the current literature.

Adjacent structured care literatures establish that food tracking can occur in supervised settings and can serve multiple clinical functions. In eating disorder higher levels of care, patient self-monitoring with clinician review has been implemented and its engagement patterns characterized across inpatient, residential, and partial hospital contexts (14). In acute hospital settings, patient dietary logging has been used primarily to generate structured nutritional reports for staff clinical decision-making rather than as a patient skill-building exercise (15, 16). In long-term care, proxy capture of dietary intake by staff rather than resident self-logging has been the predominant model, reflecting assumptions about resident capacity and workflow constraints (17). In geriatric inpatient rehabilitation, self-logging has been implemented with age-adapted interfaces and substantial structured onboarding, demonstrating feasibility in populations where digital literacy and cognitive load were identified as implementation barriers (18, 19). The variation in these adjacent literatures on participant food tracking show that tracking responsibility is not uniformly assigned to the patient and that the function tracking serves varies considerably by setting and clinical purpose.

Epilepsy KDT provides the closest implementation precedent for KMT delivery in psychiatry. International recommendations for both pediatric and adult epilepsy KDT describe food intake recording as a standard monitoring component during diet introduction, with higher participant involvement in food tracking determined primarily by diet variant and individualized to patient circumstances (12, 13). In pediatric implementation of a classic KD, food tracking is frequently executed by caregivers with dietitian support, while adult epilepsy practice more commonly uses less restrictive diet variants such as the modified Atkins diet, which requires carbohydrate monitoring using household measures rather than gram-scale weighing (13). The criteria informing tracking intensity decisions in epilepsy KDT have not been evaluated in psychiatric populations, and it cannot be assumed that delivery and tracking approaches developed for epilepsy transfer optimally to adults managing KMT amid predominant psychiatric symptoms and variable self-management capacity.

Psychiatric KMT has features of a complex nonpharmacologic intervention in that its implementation requires new behaviors from both those delivering and those receiving care. The psychosocial and behavioral dimensions of KMT implementation have been recognized in both epilepsy KDT and psychiatric KMT literatures. In epilepsy KDT, barriers and facilitators to dietary adherence have been organized using the COM-B behavior change framework, with findings extending across psychological capability, motivation, and social opportunity domains (20). In psychiatric KMT, COM-B has similarly been applied to characterize adherence-relevant factors in bipolar disorder trials (21). Reporting guidance for interventions with these features includes the Template for Intervention Description and Replication (TIDieR) (22, 23), the CONSORT extension for nonpharmacologic treatments (CONSORT-NPT) (24) and CONSORT-SPI 2018 which aligns with updated guidance for social and psychological interventions (25). This guidance requires documentation not only of what was delivered by providers but of the extent to which interventions were taken up by participants (25, 26). These standards therefore require that published psychiatric KMT studies specify what tracking instruction was given, what clinical or research staff role delivered that instruction, what methods participants were expected to use, who reviewed participant-generated dietary records and whether the records informed clinical action, and what level of independent dietary execution was expected of participants. Current psychiatric KMT studies do not meet this standard consistently. Incomplete reporting of intervention components limits replication, compromises evidence synthesis, and prevents clinicians from inferring what level of participant implementation is necessary for benefit and possibly even consistency of outcomes (27). This current reporting gap may also reflect a more fundamental ambiguity in how participant food tracking is understood as a delivery component.

Participant food tracking in psychiatric KMT is often treated as a simple implementation choice when it may represent several distinct clinical functions with different implications for how it is taught, supported, and evaluated. Whether tracking primarily serves the clinician as a data collection tool, the patient as a behavioral activation task, or the treatment relationship as food data that informs diet precision or records adherence will shape the clinical design of the intervention. Instruction requirements, outcome measurement, and the short-versus-long-term goal orientation all depend on which function participant food tracking is intended to serve. Without a conceptual framework that distinguishes these functions and the clinical decisions that surround them, research cannot determine which aspects of tracking matter, for whom, and under what conditions.

**Figure 1:**
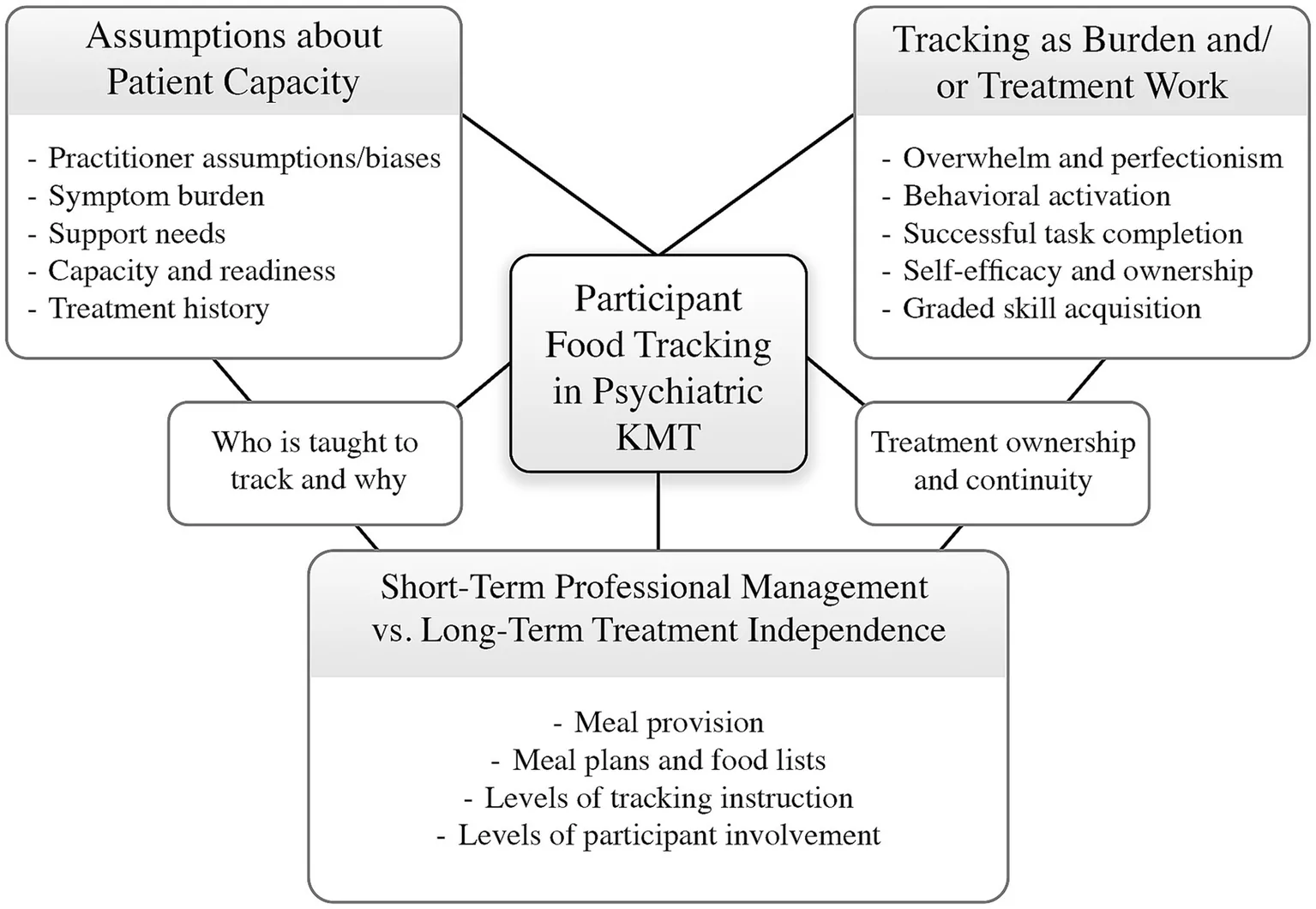
Conceptual framework for participant food tracking in psychiatric ketogenic metabolic therapy. Decisions about whether and how participants are taught to track dietary intake in psychiatric KMT are shaped by assumptions about patient capacity, the dual potential of tracking as burden and/or treatment work, and the relationship between short-term professional management and long-term treatment independence. Each of the three connects directly to the central construct, and each generates an empirical question that has not been examined in the published literature. Who is taught to track and why is the question that emerges from capacity assumptions. It bridges into decisions about short-term professional management or long-term treatment independence because those decisions rest in part on what providers believe a participant can do. Treatment ownership and continuity is the question that emerges from the dual potential of tracking as burden and/or treatment work. It bridges into the same decisions because whether tracking is experienced as burden or as treatment work shapes whether the participant develops the ownership needed to sustain KMT. Capacity assumptions and the dual potential of tracking as burden and/or treatment work are not directly connected in the framework. They shape participant food tracking in psychiatric KMT independently and through different evidence bases. The framework is hypothesis-generating and does not imply a causal sequence among the three.

This conceptual analysis develops such a framework. Published psychiatric KMT studies, epilepsy KDT implementation evidence, adjacent structured care literatures, and reporting guidance for complex nonpharmacologic interventions were synthesized to examine variation in participant tracking practices and their clinical implications. These bodies of work were integrated by mapping delivery variation across a proposed continuum of participant involvement and organizing clinical implications across three unresolved clinical questions: what assumptions about patient capacity drive decisions about whether and how tracking is taught; whether tracking functions primarily as burden or as treatment work, and what that distinction means for clinical design; and how the relationship between short-term professional management and long-term treatment independence shapes the role of participant food tracking across continuity of care (Figure 1). This synthesis is hypothesis generating and did not use systematic review methods.

## 2 Variation in participant food tracking across the published psychiatric KMT literature

Published psychiatric KMT clinical studies and program evaluations describe delivery models ranging from full meal provision with no participant tracking responsibility to outpatient self-management in which participants are taught to track macronutrient intake, with considerable variation in between (Table 1). Published case reports and case series document similar variation across a wider range of delivery contexts (Table 2). Narrative descriptions of each publication are provided (Supplementary Table 1) and state what each publication reported about delivery and participant food tracking, providing the basis for the categorical assignments in Tables 1, 2. What the published literature does not describe with consistent precision is what dietary implementation responsibilities participants carried and what level of independent dietary management was intended across the course of care.

**Table 1:** Reported variation in psychiatric KMT delivery and participant food tracking in clinical studies and program evaluations.

Study/design	Population and care context	KMT delivery	Participant tracking
Pilot study (87)	10 female inpatients with chronic schizophrenia	Professionally managed inpatient delivery. Inpatient ketogenic diet added to ongoing treatment.	Participant food tracking not specified
Open label pilot study (88)	5 weight recovered adults with anorexia nervosa and persistent eating disorder thoughts and behaviors	Intensive initiation with home continuation. Two day immersion, then home maintenance.	Participant food tracking not specified
Retrospective analysis of clinical care (6)	31 adults with severe, persistent mental illness in a psychiatric inpatient setting	Professionally managed inpatient delivery. Dietitian approved meals prepared in hospital, approved foods list, psychiatrist dietary support.	Described in part, daily food journals
Feasibility pilot study (21)	26 euthymic adults with bipolar disorder in an outpatient setting	Supported outpatient self-management. Individualized prescriptions, weekly remote RD support.	Described in part, 3-day food diary before diet initiation
Mixed-methods process evaluation (21, 89)	15 of 26 euthymic adults with bipolar disorder interviewed 1–2 months post-intervention; 4 research clinicians interviewed	Supported outpatient self-management. Individualized dietary prescriptions, weekly dietitian-led diet review meetings, tailored recipes, behavioral support strategies including goal setting and action planning, psychiatrist available as needed.	Described in part, participants calculated fat and carbohydrate ratios for meal preparation with dietitian support
Pilot trial (7)	23 adults with bipolar disorder or schizophrenia in an outpatient setting	Supported outpatient self-management. One hour teaching session, educational handouts, cookbooks, recipes, personal coach, and regular physician monitoring over 4 months.	Described in part, participants were instructed to reduce and monitor carbohydrate intake to about 20 g/day
Pilot study (90)	24 college students with major depressive disorder in an outpatient setting	Partially provisioned outpatient delivery. Dietary education session before baseline testing, extensive personalized education and ongoing support from the dietetic team via messaging app. Ten prepackaged ketogenic meals provided, then home implementation with participant selected foods and meals, plus selected shelf stable ketogenic foods.	Participant food tracking not specified
Retrospective evaluation (91)	19 adults with varied mental health diagnoses, including anxiety, bipolar disorder, depression, ADHD, autism, schizophrenia spectrum disorders, and premenstrual dysphoric disorder (PMDD), enrolled in a remotely delivered online program	Group based supported self-management. Practitioner led online program with personalized macronutrient targets, 8 h of education over 8 weeks, then weekly 1 h support and Q&A sessions. Participants prepared their own meals using a ketogenic food list.	Reported, app-based macronutrient tracking
Open-label pilot feasibility trial (8)	11 adults with moderate to severe MDD or bipolar depression enrolled in a remotely delivered program	Intensive initiation with home continuation. Dietitian-supervised two-week induction phase followed by 12-week home maintenance with weekly dietitian support.	Described in part, daily food records reviewed weekly by dietitian
Randomized clinical trial (9)	100 adults aged 18–65 with treatment-resistant depression in an outpatient setting	Fully provisioned outpatient delivery. Pre-prepared ketogenic meals and snacks provided, weekly 30-min dietitian counselling calls, and written dietary materials. Participants who declined provided meals received written resources for home meal preparation.	Participant food tracking not specified
Single-arm pilot study (92)	20 adults with binge-eating disorder	Supported outpatient self-management. Structured KD delivered by a registered study dietitian trained in ketogenic therapy. Two initial 60-min educational sessions; weekly then bi-weekly support calls. Personalized meal plans, recipes, and grocery lists provided.	Reported, daily food records
Single-arm feasibility trial (93)	22 adult women with anorexia nervosa in an outpatient setting	Partially provisioned outpatient delivery. Fourteen-week weight-maintaining ketogenic therapy introduced by study dietitian, with optional ketogenic meal delivery or self-prepared meals under dietitian guidance, weekly psychiatrist and dietitian meetings, and optional peer counseling.	Described in part, post-meal photographs submitted for dietitian review as needed

KMT delivery was grouped into broad descriptive categories to support comparison across studies while avoiding overinterpretation of underreported methods. Categories were assigned only when the publication explicitly described who managed day-to-day implementation and how food was provided. Professionally managed inpatient delivery refers to institutionally prepared or tightly controlled meals in an inpatient setting. Intensive initiation with home continuation refers to a structured initiation phase followed by home implementation. Fully provisioned outpatient delivery refers to outpatient models in which pre-prepared meals were provided to participants as the default mode of implementation. Supported outpatient self-management refers to outpatient delivery in which participants selected or prepared food with ongoing clinical support. Partially provisioned outpatient delivery refers to outpatient models in which some meals or foods were provided while participants managed other aspects of implementation. Group based supported self-management refers to education centered models in which participants implemented KMT in daily life with group or program support. Delivery model not sufficiently specified was used when the publication did not describe the delivery approach in enough detail to permit a more specific designation. Participant food tracking was coded as reported, described in part, or not specified, based only on what was clearly stated in the publication.

**Table 2:** Reported variation in psychiatric KMT delivery and participant food tracking in case reports and case series.

Study/design	Population and care context	KMT delivery	Participant tracking
Letter to the editor, single case report (94)	One 49-year-old woman with severe treatment resistant rapid cycling bipolar illness	Supported outpatient self-management. Patient self-managed a classic ketogenic diet followed by a modified ketogenic diet using MCT oil, with clinical team oversight implied but not detailed.	Participant food tracking not specified
Case series (95)	Two adult women with type II bipolar disorder	Supported outpatient self-management. Participants initiated and maintained ketogenic diet in outpatient settings. Foods consumed were described by case. Diet supervision, educational support, and meal provision not specified.	Participant food tracking not specified
Letter to the editor, case studies (96)	Two adults with schizoaffective disorder	Delivery model not sufficiently specified. Ketogenic diet described at case level with example foods.	Participant food tracking not specified
Case studies (97)	Two adult women with schizophrenia treated in an outpatient setting	Supported outpatient self-management. Participants self-prepared ketogenic meals independently; details about clinical supervision or educational support were not described.	Participant food tracking not specified
Case series (98)	Three adults with obesity and self-reported binge eating and food addiction symptoms	Supported outpatient self-management. Outpatient obesity medicine physicians taught a low-carbohydrate ketogenic diet with carbohydrate restriction to 20 to 30 g/day, whole-food guidance, food lists, and regular follow-up visits. Meal provision not described.	Participant food tracking reported, dietary recall questionnaires and carbohydrate tracking
Case series (99)	Three adults with severe anorexia nervosa	Delivery model not sufficiently specified. Patients self-adopted ketogenic diets following online educational materials, internet readings, social media recommendations, or internet research; one patient was later followed by a nutritionist specializing in ketogenic diets.	Participant food tracking not specified
Case report (100)	A 53-year-old woman with treatment-resistant bipolar II disorder	Supported outpatient self-management. Ketogenic diet initiated remotely with individualized macronutrient targets, regular virtual meetings, and dietary education with ketogenic professional.	Reported, macronutrient tracking with app
Case series (101)	Three adults aged 32–36 with major depression, generalized anxiety disorder, and complex psychiatric comorbidities	Supported outpatient self-management. Ketogenic metabolic therapy initiated remotely with dietitian-supervised support including individualized macronutrient targets, dietary education, virtual group sessions, regular clinician contact, and community support.	Reported, daily photo journaling
Retrospective case report (102)	Adult woman with PTSD, ADHD, binge-eating disorder, bipolar II disorder, depression, anxiety, and premenstrual dysphoric disorder	Group based supported self-management. Remote KMT psychoeducation, twice-weekly professional and peer support calls, and private community support.	Reported, macronutrient tracking with app
Retrospective case study (103)	A 47-year-old woman with chronic treatment-resistant major depressive disorder	Group based supported self-management. Online KMT program with self-paced education and group sessions with a ketogenic professional.	Reported, macronutrient tracking with app
Case report (104)	A 37-year-old woman with obsessive-compulsive disorder and ulcerative colitis	Supported outpatient self-management. Personalized whole-food ketogenic diet developed by a ketogenic registered dietitian, implemented at home, with twice-weekly dietitian visits for 1 month then weekly visits.	Reported, photo journaling of food intake
Retrospective case series (105)	Two females, ages 17 and 32, with schizoaffective disorder	Supported outpatient self-management. Modified ketogenic diet initiated and monitored by a ketogenic professional with regular virtual KMT support meetings.	Reported, macronutrient tracking with app
Case series (5)	Three adults with obsessive-compulsive disorder	Delivery model not sufficiently specified. Ketogenic diets adopted in outpatient settings; foods consumed were described in some cases.	Participant food tracking not specified
Retrospective case report (11)	A 26-year-old man with treatment-resistant obsessive-compulsive disorder	Delivery model not sufficiently specified. Self-treated modified ketogenic diet implemented at home; no medical oversight or dietary professional guidance.	Reported, daily dietary intake tracking using a personal spreadsheet
Case report (28)	A 48-year-old woman with treatment-resistant schizophrenia and metabolic syndrome	Professionally managed inpatient delivery. Medical ketogenic diet in a hospital setting with meals planned by the hospital dietary department, delivered to the patient’s room, and monitored by hospital staff.	Participant food tracking not specified
Case report (106)	A 32-year-old man with schizophrenia	Supported outpatient self-management. Carnivore ketogenic diet implemented at home with nutritional therapy practitioner support to optimize meal pattern and fat intake; later self-managed.	Participant food tracking not specified
Retrospective case report (10)	A 45-year-old woman with treatment-resistant post-traumatic stress disorder	Supported outpatient self-management. Remote KMT with an initial one-hour consultation followed by structured individual virtual sessions with a ketogenic professional.	Participant food tracking not specified
Case report (107)	A 34-year-old woman with bipolar II, comorbid personality disorder and alcohol use disorder in outpatient psychiatric care	Supported outpatient self-management. Three-month modified ketogenic diet implemented at home after ketogenic diet education, with psychiatric follow-up and no dietitian follow-up.	Participant food tracking not specified

KMT delivery was grouped into broad descriptive categories to support comparison across case reports and case series while avoiding overinterpretation of under described methods. Categories were assigned only when the publication described who managed day-to-day implementation and how food was provided. Professionally managed inpatient delivery refers to institutionally prepared or tightly controlled meals in an inpatient setting. Intensive initiation with home continuation refers to a structured initiation phase followed by home implementation. Supported outpatient self-management refers to outpatient delivery in which participants selected or prepared food with ongoing clinical support. Partially provisioned outpatient delivery refers to outpatient models in which some meals or foods were provided while participants managed other aspects of implementation. Group based supported self-management refers to education centered models in which participants implemented KMT in daily life with group or program support. Delivery model not sufficiently specified was used when the publication did not describe the delivery approach in enough detail to permit a more specific designation. Participant food tracking was coded as reported, described in part, or not specified, based only on what was stated in the publication.

In many studies and case reports, participant food tracking may be present as a recorded behavior without being described clearly enough to determine whether it functioned as self-monitoring, clinician-guided adherence assessment, skill acquisition, or some combination of these. The criteria by which practitioners determine tracking instruction intensity are not documented in the published literature reviewed here. The variation itself implies that active decisions about participant food tracking involvement are being made, but whether those decisions reflect assumptions about patient capacity, available clinical resources, philosophical orientation toward participant skill acquisition, or some combination of these factors remains unexamined. Patients who appear most disabled by psychiatric symptoms may nonetheless demonstrate strong motivation and ability to acquire dietary self-management skills when given the opportunity. Caregiver and clinician assumptions about patient capacity have been identified as influences on dietary therapy delivery decisions in adjacent ketogenic diet literatures, yet whether those assumptions accurately reflect patient capability remains unexamined (20). Whether burden-reduction assumptions built into professionally managed KMT delivery models serve patients well over the longer term has not been evaluated in the psychiatric KMT literature.

## 3 What counts as participant food tracking in this paper

**Figure 2:**
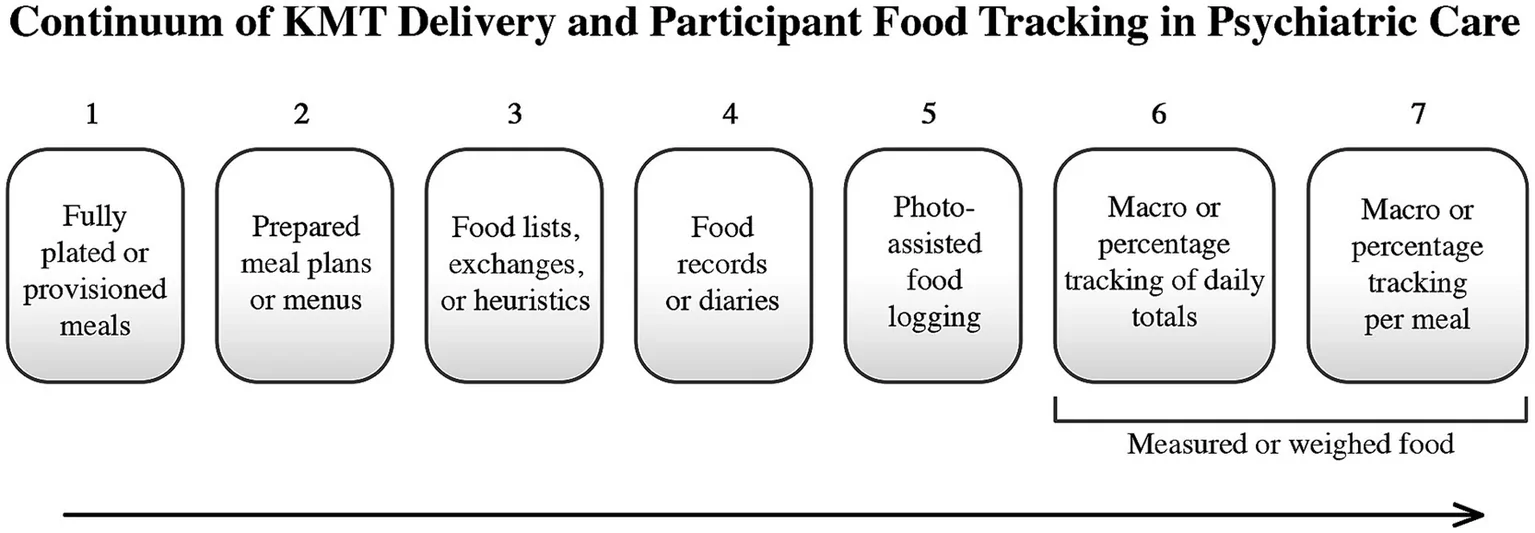
Continuum of participant involvement in psychiatric KMT dietary implementation and food tracking. The figure depicts a continuum of increasing participant involvement in psychiatric KMT dietary implementation. The left side represents professionally managed delivery approaches in which food intake is controlled or structured by the treatment setting, dietitian, clinician, meal service, or prepared meal plan. Movement across the continuum reflects increasing participant responsibility for selecting, preparing, quantifying, and recording dietary intake. Placement on the continuum is based on these reported participant tasks. Food lists, exchanges, meal heuristics, and meal plans may increase participant involvement in implementation but do not necessarily require participant-generated dietary records. Participant food tracking is represented only at points on the continuum in which the participant generates a record of dietary intake. Estimated food tracking and weighed food tracking are distinguished because they require different participant tasks and produce records with different levels of dietary precision. The continuum is descriptive and does not rank delivery approaches, imply that greater participant involvement is preferable, or treat all forms of dietary implementation support as participant food tracking.

In clinical practice, participant food tracking and the instruction that surrounds it are often experienced as one continuous activity. The narrower definition isolates participant-generated dietary recording so that decisions about tracking can be studied as a delivery component with its own clinical rationale. Not all approaches described as food tracking in the psychiatric KMT literature require participants to generate a record of their own dietary intake. Fully provisioned meals and prepared meal plans place dietary implementation responsibility with the clinician, dietitian, or delivery system (6, 9, 28). Food lists, exchange systems, and meal heuristics shift dietary execution to the participant but do not require a participant-generated intake record. A participant who receives fully provisioned meals, whether dietitian-prepared or supplied through a meal service, and selects from an approved food list is not performing food tracking in any operational sense, regardless of whether adherence is confirmed through ketone testing or clinical staff observe meal consumption. Participant food tracking, as used here, refers specifically to delivery approaches in which participants generate their own records of dietary intake. The range of approaches that meet this definition and their relationship to delivery models that do not are organized along a continuum of participant involvement from fully provisioned meals to food scale weighed entry of daily or per meal macronutrient ratios (Figure 2).

The nutritional epidemiology literature treats estimated and weighed food records as methodologically distinct approaches (29, 30). Estimated records quantify portions using household measures or visual aids, while weighed records require all foods to be measured on a scale before consumption (29–31). Weighed records produce more precise dietary data and serve as the reference standard against which estimated records are validated, yet the app-based dietary self-monitoring literature does not distinguish between them, which means studies reporting that participants “tracked food” cannot indicate whether the data produced were precise enough to support clinical dietary adjustment (30, 32). The epilepsy KDT clinical tradition draws a more clinically applicable distinction. International consensus guidance specifies that the classic ketogenic diet requires a food scale to weigh meals, while the modified-Atkins diet and the low glycemic index treatment do not require precise weighing of food ingredients, with this difference treated as clinically meaningful in diet selection and participant instruction (12, 13).

Weighed food tracking, as used in this conceptual analysis, refers to participant-generated intake records based on food scale measurement of quantities before logging. Estimated food tracking refers to records based on household measures, participant-estimated portion sizes entered manually, or selection of generic database entries without weighing (12, 29, 33). The precision of participant-generated data cannot be determined from the tool output alone. No standardized approach to distinguishing weighed from estimated entry as a behavioral variable has been established in the app-based self-monitoring literature, and the distinction has rarely been built into dietary recording tools as a design feature (32, 34). Because estimated and weighed participant food records require different recording tasks and produce dietary data with different levels of precision, distinguishing them may improve reporting specificity in psychiatric KMT studies (Supplementary Table 2).

Participant food tracking as defined here is distinct from several related clinical activities. Ketone testing confirms whether nutritional ketosis has been achieved but does not require the participant to record, quantify, or review dietary intake. It is an adherence verification mechanism, not a form of dietary recording. Dietary awareness, including knowledge of ketogenic foods or carbohydrate thresholds, describes what a participant knows rather than what they record. The full taxonomy of dietitian practices, including prescription design, meal planning, and clinical review of dietary data, shapes the conditions under which participant tracking occurs but is not itself the construct under examination. Participant food tracking as defined in this conceptual analysis refers to participant-generated dietary recording with the precision of that recording being described as estimated or weighed.

Use of a shared operational definition for participant food tracking has practical consequences for how the field of metabolic psychiatry can evaluate this delivery component of KMT. When published studies report that participants tracked food intake without specifying whether that involved estimated or weighed recording, whether daily or per-meal macronutrient totals were expected, level of participant training was provided, or specificity regarding who reviewed the data and how often, the resulting evidence cannot be compared across studies or used to inform clinical decisions (32). Coherent research questions about the relationship between participant food tracking and treatment persistence, burden, clinical outcomes, and post-support dietary continuation require the kind of operational specificity the field has not yet applied to this construct.

## 4 Assumptions about patient capacity

Decisions about whether participants in psychiatric KMT are taught to track their dietary intake, and at what level of precision, may determine where on the continuum of participant involvement they are placed, how much tracking instruction they receive, how much support they are given, what capacity for independence they are building, and how likely they are to continue the diet after professional support has ended. The criteria by which practitioners determine whether and how tracking is taught have not been described, and the assumptions driving those decisions remain unexamined.

The assumption that serious psychiatric illness precludes the acquisition of complex self-management skills is not supported by the adjacent evidence base. A meta-review of 11 literature reviews found that up to three-quarters of psychiatric patients, including individuals with schizophrenia and bipolar disorder, may have capacity to make medical decisions in the context of their illness, and that decisional capacity impairments in psychotic patients, when present, are temporal, identifiable, and responsive to information-enhancing interventions such as simplification, training, and shared decision-making (35) Cognitive remediation research offers particularly direct evidence against this assumption. A meta-analysis of 130 randomized clinical trials found that patients with fewer years of education showed larger gains in cognition and functioning after cognitive remediation, while higher baseline symptom severity predicted larger cognitive gains and lower premorbid IQ predicted larger functional gains (36). Supported employment research reinforces this pattern. A meta-analysis found that Individual Placement and Support improved competitive employment outcomes relative to control conditions across subgroups of people with mental disorders, although employment-rate effects were relatively stronger in severe mental illness, schizophrenia-spectrum samples, and lower-symptom-severity groups, and were not significantly moderated by illness duration or level of functioning (37). Across decisional capacity, cognitive remediation, and supported employment, the adjacent evidence base shows that adults with serious psychiatric illness can acquire complex skills when given the conditions that support them. The assumption that psychiatric patients cannot acquire the skills required for food tracking has not been tested in the psychiatric KMT literature, and the adjacent evidence base does not justify treating it as established.

Mental health practice has historically been organized around the expectation that serious mental illness follows a deteriorating or maintenance course, with systems oriented toward symptom management and relapse prevention rather than the development of patient self-management capacity (38). This pattern persists in contemporary clinical settings, where practitioners may inadvertently perpetuate hierarchical and paternalistic practices that question individuals’ capacities to recover (39). Clinical experience with acutely ill or chronically hospitalized populations has been identified as a source of systematically pessimistic beliefs about patient capability, with exposure to the most severely affected patients producing distorted estimates of what recovery is possible (40). Provider pessimism about recovery is a documented source of stigma in healthcare, manifesting as low treatment expectations and paternalistic approaches that assume incapacity rather than assess it, with patients describing these encounters as experiences of being excluded from decisions and treated in dismissive or demeaning ways (41). In mental health settings, therapeutic pessimism has been defined as the inclination to believe that people with mental illness are difficult to treat or immune to treatment, with research establishing an association between this pessimism and professional helplessness that contributes to inadequate treatment provision (42). Protective attitudes among providers have been specifically identified as hampering positive risk-taking and personal development, with overprotectiveness documented as a direct contributor to under-referral to vocational and skill-building services (43). Among practitioners in assertive community treatment settings, explicit and implicit stigma toward serious mental illness was associated with lower recovery orientation (44), with low-recovery-orientation teams characterized as more paternalistic and more focused on patient limitations than strengths (45). These documented practitioner attitudes are worth examining in psychiatric KMT delivery, where similar assumptions could shape decisions about who is offered food tracking instruction and at what level of involvement without being recognized.

Assumptions about patient capacity may also reflect concerns about treatment retention. Practitioners who anticipate that food tracking demands will reduce engagement or accelerate dropout may simplify delivery rather than teach tracking skills, whether or not that concern is warranted. In a feasibility study of dietary assessment methods for research, adults with schizophrenia and related disorders completed food diaries, weighed records, and photo records at completion rates in the 70–80% range when provided equipment, detailed instructions, and investigator support (46). The study evaluated dietary recording for research purposes rather than as a therapeutic self-management activity, and whether similar feasibility extends to participant food tracking as a sustained activity in psychiatric KMT has not been examined (46). Provider expectations of patients with schizophrenia are significantly lower than expectations of otherwise identical patients without the diagnosis, including expectations of treatment adherence, capacity to manage health-related tasks, and ability to understand educational materials (47), and provider willingness to support self-management during hospitalization is consistently conditional on capacity and adherence judgments (48). The field of metabolic psychiatry has not examined whether decisions about who is taught to track in psychiatric KMT reflect demonstrated participant capacity or untested practitioner expectations about participant capacity for self-management.

## 5 Tracking as burden and treatment work in adjacent literatures

Participant food tracking in psychiatric KMT may function as a source of burden that undermines treatment engagement, as a form of treatment work that builds self-management capacity, or as both simultaneously within the same task. No published psychiatric KMT study has examined which of these descriptions applies, under what conditions, or for whom. The decision to teach or not teach tracking implicitly assumes an answer to this question, yet the assumption remains untested.

Whether dietary self-monitoring triggers perfectionistic distress in psychiatric populations has not been tested. The evidence on this question comes primarily from non-clinical and eating-disorder populations specifically using commercial applications without clinical supervision, a context that differs materially from clinician-supervised macronutrient tracking in psychiatric KMT. In a non-clinical sample of college women newly introduced to fitness tracking, a formal moderator test found no significant interaction for perfectionism, though rumination emerged as a significant moderator of weight and shape concerns (49). Randomized introduction of dietary self-monitoring to previously naive users screened to be at low risk for eating disorders produced no adverse psychological effects over one month, though the authors acknowledged that null results may reflect the selection of a low-risk sample rather than absence of risk in higher-risk groups (50). The qualitative literature documenting rigid and distressing tracking experiences comes largely from these same non-clinical college populations (51). In contrast, a population-based sample of young adults followed for four years showed that using weight-related self-monitoring apps with a weight-management motivation was associated with an increase in disordered weight control behaviors, while tracking with a physical-activity motivation was not (52). Baseline risk profile, duration, and the motivation and structure of tracking may be meaningful moderators of tracking-related risk rather than tracking itself being inherently harmful or safe.

A retrospective study of individuals with diagnosed eating disorders found that approximately three quarters had used a food tracking app to track calories, and 73% of users perceived the app as having at least somewhat contributed to their eating disorder. Perceived contribution correlated with eating disorder symptom severity on measures of restraint, weight concern, and shape concern (53). The authors acknowledged directly that the retrospective self-report design cannot establish whether app use contributed to disorder onset or whether individuals with more severe symptoms were more likely to engage with calorie tracking. The sample was primarily individuals with anorexia nervosa recently discharged from residential or partial hospitalization treatment. Most were in ongoing outpatient care using a commercial app not designed for eating disorder populations and without clinical direction for its use in that context. The findings describe what calorie tracking looked like for individuals with active eating disorders using an unsupervised commercial app, and they do not establish what macronutrient tracking would look like under clinician supervision in psychiatric KMT.

The relationship between dietary self-monitoring and clinical outcomes may be bidirectional, in that more frequent tracking may support better outcomes while poorer outcomes may also reduce the participant’s engagement with tracking. A randomized behavioral weight loss trial found that more frequent self-monitoring predicted greater weight loss within the same month across all conditions, and in the group-based treatment arm, poorer weight loss in one month predicted reduced self-monitoring the following month (54). The authors interpreted this pattern as specific to the group arm in which participants attended weekly groups together and tracked under shared accountability to other members. When weight loss stalled for participants in the group arm, shared accountability eroded and tracking engagement dropped. The authors speculated that participants with poor weight loss may have experienced shame in front of fellow participants and interventionists, and may have avoided self-monitoring because logging reminded them of poor outcomes associated with unpleasant emotions. Whether the same reverse effect operates in psychiatric KMT has not been examined, and the question carries weight specific to this context. Tracking in psychiatric KMT may be not only a behavior-change support but an activity through which the clinician refines diet composition and troubleshoots whether the prescription needs adjustment, adherence is incomplete, or the participant is not responding to a correctly implemented diet. If a participant becomes discouraged by what they perceive as non-response in specific contexts and reduces tracking engagement, the clinician loses the data needed to troubleshoot and adjust treatment at the point it is most needed.

Qualitative evidence in adults with serious mental illness documents dietary change as a source of both self-efficacy and distress, with participants describing agency and guilt in relation to the same dietary behaviors (46, 55). In semi-structured interviews with 28 adults living with schizophrenia spectrum disorders, first-episode psychosis, bipolar disorder, or major depressive disorder, participants described cooking for themselves and eating healthily as producing personal agency and mood improvement, alongside accounts of guilt and self-directed frustration following dietary choices that departed from their intentions (55). The authors characterized this as an inner conflict in which positive and negative dimensions of the same behaviors coexisted in tension rather than alternating sequentially. Weight and body concerns drove much of the negative experience but did not displace the positive accounts participants also described.

The potential burden of dietary recording in this population was further examined in a related feasibility study of dietary assessment methods (46). Among 63 adults with SMI completing photographic food records, written food diaries, and weighed food records across two assessment periods, the food diary and photographic record were reported as acceptable and feasible methods for dietary assessment. At the same time, participants reported self-perceived stigma when using monitoring equipment in the presence of others, and five participants reported under-eating during the assessment period as a direct consequence of recording their intake. Burden of documentation was cited as a reason for dropout (46). These findings come from a research monitoring protocol rather than a therapeutic ketogenic intervention. They also reflect responses to specific demands made within research monitoring protocols, including requirements to record intake across social situations, rather than responses to food tracking itself. The clinical design of how tracking is asked of the participant is itself a variable that psychological evidence can inform.

Behavioral weight loss research has shown that dietary self-monitoring declines over time. Fewer than half of participants in one study sustained tracking beyond week nine to ten across a calorie counting application, a wearable bite counter, and a photographic meal application (56). A similar pattern was observed in time spent monitoring across a 24-week behavioral weight loss intervention, with participants spending an average of 23.2 min per day during the first month and 14.6 min per day by month six, which the authors attributed not to participant burden alone but to growing efficiency as participants learned the system and accumulated a repertoire of commonly consumed foods (57). Consistent early monitoring, rather than recording in precise detail or for an indefinite time frame, may be more important for sustained behavior change. Reductions in self-monitoring intensity over the course of an intervention may therefore reflect dietary learning and behavioral internalization rather than disengagement.

Self-management skill acquisition produces measurable gains in adults with serious mental illness. A systematic review and meta-analysis of 37 randomized controlled trials found that self-management interventions improved symptoms, functioning, and self-efficacy at end of treatment. Empowerment and hope did not improve at end of treatment but emerged at follow-up (58). Studies measuring outcomes only at end of treatment would not capture these benefits without a similar design of research participant follow-up. In qualitative research with adults with serious mental illness using the Wellness Recovery Action Plan framework, an autonomy-supportive environment fostered a progression from externally supported learning to autonomous application of self-management strategies. Participants described an internal shift in volition and personal responsibility that went beyond skills acquisition alone (59). Self-monitoring with feedback, graded tasks, and professional human support are among the most effective behavior change techniques in digital health interventions for the prevention and management of noncommunicable diseases (60). Scaffolding, in which structured early support is gradually reduced as competence develops and the participant takes on increasing responsibility over time, is one framework through which participant food tracking instruction could build self-management capacity rather than remain an external demand.

Recovery-oriented service delivery for adults with serious mental illness prioritizes participant agency and skill development, including the setting of personally meaningful goals, over professional management of the illness, and across 40 studies of recovery services the active engagement of participants in their own care were associated with measurable gains in self-efficacy and functioning alongside improvements in quality of life (61). The largest controlled test of a strengths-based case management model for serious mental illness enrolled 1,276 participants, 81% of whom carried a schizophrenia-spectrum diagnosis, and found that strengths-based support produced significant self-efficacy gains, with participants setting more personally meaningful goals and using professional contact more selectively rather than more broadly over the course of care (62). These findings come from recovery-oriented services in serious mental illness populations rather than from psychiatric KMT specifically. Together these findings may support a design principle for tracking instruction in psychiatric KMT, one in which clinical scaffolding reduces as participant competence develops and the participant is treated as the agent of their own dietary skill acquisition, consistent with the recovery-oriented approach that has produced self-efficacy gains in adjacent populations.

Self-efficacy theory identifies mastery experiences as the most potent source of perceived capability, with successful performance of a task building confidence not only for that task but across behavioral domains (63). In a qualitative study of illness self-management in adults with schizophrenia spectrum disorders, bipolar disorder, major depression with psychotic features, and PTSD, all with co-occurring chronic medical conditions, participants who practiced weekly self-management action plans in real-world settings and received structured group feedback described a lasting shift in how they approached their health and recovery (64). Participants reported an increased sense of control and confidence that extended beyond the specific behaviors practiced to a holistic belief in their capacity to pursue goals, solve problems, and persist through setbacks in multiple areas of their lives. The authors identified this shift as consistent with mastery-driven self-efficacy enhancement as described by Bandura (63).

In a study of daily dietary self-regulation, successful adherence to dietary goals on a given day predicted increased self-regulatory effort the following day, and this effect was strongest in individuals who perceived themselves as unsuccessful dieters, the group least confident in their own capability and most responsive to the experience of success (65). In a 12-week standalone digital weight loss intervention emphasizing daily self-monitoring, self-efficacy for tracking dietary intake declined over the course of the intervention. Participants whose tracking self-efficacy improved in the first month were the most likely to sustain engagement and achieve weight loss outcomes (66). Participant food tracking is a repeated daily task in which successful completion is immediately visible to the participant. If the self-efficacy mechanism identified in adjacent populations operates in this context, then the level of participant involvement in food tracking in psychiatric KMT may be instrumental in the development of self-efficacy that extends beyond dietary management to the participant’s broader sense of personal capability and agency in their own care.

Behavioral activation is an established therapeutic approach for depression in which structured engagement in daily activities reverses the withdrawal and avoidance cycles characteristic of the condition (67, 68). The therapeutic effect of behavioral activation comes from the activity itself rather than from the insight or data it produces. Three independent qualitative studies describe activation-consistent mechanisms in adults completing structured daily monitoring. Daily app-based self-monitoring instilled routine and prompted engagement in a subset of adults with depression who lacked regular daily structure, with participants in that subset describing commitment to activity before each scheduled measurement prompt, despite null effects on standard symptom outcomes across the full sample (69). Completion of daily symptom entries on a standalone automated monitoring app without active human support was described by adults with psychosis as rewarding and linked to a sense of achievement, with participants reporting that the recording task itself generated motivation to engage in daily activity (70). Over half of adults with bipolar disorder who completed daily mood and activity monitoring reported that the monitoring prompted behavioral change, with most increasing their exercise levels and some describing improved sleep hygiene, a behavior change in a domain that was not itself being tracked (71). Participant food tracking in psychiatric KMT is a daily structured task that may provide additional treatment work in the form of behavioral activation.

The burden and treatment work evidence comes from behavioral weight loss, chronic disease self-management, and digital health intervention contexts rather than from therapeutic dietary interventions for psychiatric conditions. Psychiatric KMT involves a dietary pattern maintained for therapeutic carbohydrate restriction or macronutrient ratios rather than caloric restriction for weight loss, and the self-management demands of maintaining therapeutic ketosis may differ from those documented in the behavioral weight loss literature. Maintaining therapeutic ketosis while managing psychiatric symptoms that affect cognition and motivation may involve distinct self-management demands, but whether and how those demands differ is an empirical question. What participants are taught, how tracking is supported, and what level of independence is intended are not consistently described in the published psychiatric KMT literature, and without that information the relationship between participant food tracking and treatment outcomes cannot be meaningfully studied.

## 6 Short-term professional management versus long-term treatment independence

Psychiatric KMT delivery models differ in what dietary implementation skills participants are expected to acquire during treatment and carry forward after professional support has decreased or ended. Some models provide dietary education, food selection guidance, and professionally managed meal composition without teaching participants to measure, record, or independently monitor macronutrient intake. Others teach food measurement and daily macronutrient tracking as core treatment skills. These approaches reflect different definitions of what constitutes sufficient preparation for independent dietary continuation, and they may be combined or sequenced within a single delivery model. Whether different levels of participant involvement in food tracking produce different rates of dietary continuation after professional support ends has not been examined in the published psychiatric KMT literature.

Adherence to ketogenic dietary therapy in epilepsy declines progressively across all diet variants, with mean retention rates falling from 79.7% within the first six months to 66.7% after one year, 46.5% after two years, and 37.7% at three years and beyond (72). Documented reasons for discontinuation include inefficacy in seizure control, adverse effects, dietary restrictiveness, difficulty preparing meals, and cost. This decline occurs despite ongoing clinical contact and across populations receiving the classic ketogenic diet, modified Atkins diet, and medium-chain triglyceride diet (72). Across health behavior change interventions more broadly, no completed adaptive stepped-care study has tested reducing professional support for treatment responders in any population (73).

Sustained therapeutic ketosis requires dietary precision, and the available evidence from adjacent ketogenic diet populations shows that dietary continuation and dietary precision diverge when professional support decreases (74, 75). In a ketogenic diet trial for adults with relapsing multiple sclerosis, 91% of participants intended to continue at study completion, yet only 21% maintained the level of carbohydrate restriction specified in the protocol three months later, and whether participants continued any form of self-monitoring was not reported (75). A two-year remote care intervention for adults with type 2 diabetes provided continuous health coaching and monitored dietary adherence through biomarker testing rather than participant-generated dietary records. Participants were instructed to restrict carbohydrates to below 30 grams per day and achieve nutritional ketosis, and how participants were taught to estimate their own carbohydrate intake was not described. Retention at two years was 74%, but only 14.1% achieved blood ketone levels consistent with nutritional ketosis at the two-year laboratory measurement (74). In behavioral weight loss programs, dietary self-monitoring declined earlier and more steeply than other self-monitoring behaviors, with only 21% of participants maintaining consistent food logging during reduced-contact maintenance (76). Structured professional contact delayed this decline but did not prevent it, and once participants disengaged from dietary self-monitoring, only one-third resumed (76, 77).

Initiating a new health behavior and maintaining it over time require different mechanisms, and delivery models that address only initiation may not prepare participants for the demands of long-term maintenance (78). A systematic review of 100 behavior change theories identified five mechanisms for maintenance that are distinct from those that support initiation, including motives that develop only after the behavior is adopted, self-regulation capacity under changing conditions, and habit formation through repetition in stable contexts (78). Whether a person has the capacity to perform a behavior does not ensure its enactment when symptom burden intervenes. In a study of 662 adults with chronic obstructive pulmonary disease, 14% demonstrated preserved physical capacity on exercise testing but did not meet daily activity thresholds in their own lives (79). Adults living with serious mental illness face cognitive, motivational, and functional disruptions that may produce a comparable gap between what a person has learned to do and what they can sustain under the conditions of daily life. A Delphi consensus statement on KMT for serious mental illness states that there is no evidence to suggest how long KMT may need to be continued once treatment goals have been achieved (80). If or when participant food tracking instruction begins during treatment, how precisely they are asked to track, how long it continues, and what degree of independence is intended are delivery decisions that may shape whether a participant can sustain a dietary pattern that has no established stopping point.

These delivery decisions are also shaped by the settings in which psychiatric KMT is delivered. In at least one psychiatric inpatient cooking intervention, dietary diaries were not implemented due to limited resources (81). Staffing, kitchen infrastructure, and clinical priorities may constrain what tracking instruction is feasible in a given setting, independent of what would be clinically useful. The level of participant involvement in food tracking may be a delivery decision that carries consequences for dietary precision, self-management capacity, and clinical independence after professional support ends. Treatment effects in psychiatric KMT may still be emerging at the point where professional support decreases, and a participant who cannot maintain dietary precision independently may be unable to sustain therapeutic ketosis beyond that point. How participant food tracking instruction is designed and sequenced within a delivery model may shape whether a participant can independently continue a treatment that may need to last well beyond the period of professional support.

In psychiatric rehabilitation, improvement in illness self-management skills is directly associated with personal recovery, and clinical recovery does not appear to be a prerequisite for that association (82). The level of participant involvement in food tracking in psychiatric KMT may not be a decision made once at treatment entry. A participant’s capacity for tracking and the burden tracking imposes may both shift as treatment progresses, and the delivery decision may need to shift with the participant rather than stay fixed at treatment entry. Self-efficacy for food tracking declines for most participants during dietary interventions, and the minority whose tracking self-efficacy improves early are the participants most likely to sustain engagement and achieve outcomes (66). A participant whose tracking involvement is never reassessed may remain in a professionally managed model not because they lack ability but because the delivery model did not plan for the transition, or because the program is operating under an assumption that further skill development is unnecessary.

## 7 Implications for research and program development

Ongoing psychiatric KMT studies may advance research on participant food tracking by reporting level of participant involvement and utilizing additional measurements to investigate categories of inquiry not currently being investigated. Reporting can be refined within existing protocols to specify what tracking instruction participants received and how the resulting dietary information was reviewed within the clinical context of delivery. Additional measurements, including prospective assessment of participant burden and direct observation of clinical decision-making about tracking instruction, are not available from study reports alone and could be added to studies already planned or underway. Nested delivery condition contrasts and ecological momentary assessment are available as embedded designs and would generate the kind of within-study variation the current literature cannot provide. Comparative questions about delivery models become answerable only after the construct has been described consistently enough across studies to permit comparison (Table 3).

**Table 3:** Research priorities and candidate study designs for participant food tracking in psychiatric KMT.

Research priority/decision point	Outcomes and variables to report	Feasibility and acceptability indicators	Design and reporting priorities	Candidate study designs	Research rationale and key sources
Level of participant involvement in KMT implementation	Whether the diet is delivered through meal provision, meal plans, food lists, tracking instruction, or higher levels of participant implementation. Who manages day-to-day implementation and what level of participant involvement is intended.	Fit of the delivery model to setting, staffing and participant capacity	Specificity of delivery model to avoid collapsing all approaches into diet education or diet support that does not clarify participant level of involvement in food tracking. Professionally managed, hybrid, and participant-managed approaches should be carefully distinguished along the continuum of participant involvement (Figure 2).	Cross-sectional surveys of practitioner delivery model characteristics, comparative descriptive studies, multisite naturalistic comparisons, nested delivery condition contrasts within broader KMT studies, grounded theory, and sequential exploratory mixed methods.	Published psychiatric KMT studies range from professionally managed inpatient meal provision (6, 28) to self-directed outpatient implementation without professional dietary involvement (11, 97), but participant food tracking is not reported consistently enough to permit meaningful comparison across delivery models (Tables 1, 2).
Level of participant involvement required by delivery model	Level of participant involvement in KMT implementation, timing of food tracking instruction, support intensity, setting, patient preference, uptake of skill instruction, continuity after support changes, and independent dietary execution.	Fit of the delivery model to symptom burden, support needs, staffing, workflow, and setting.	Specificity in reporting when greater participant food tracking skill acquisition is expected, encouraged, delayed, or avoided. Distinguish clinically indicated limits from decisions shaped by staffing, workflow, or unexamined assumptions about patient capacity.	Comparative descriptive studies, pragmatic cohort comparisons, multisite naturalistic comparisons, nested delivery contrasts within broader KMT studies, grounded theory, interpretive phenomenological analysis, and sequential exploratory mixed methods.	Psychiatric KMT delivery models embed different expectations about participant involvement in dietary implementation, but published studies rarely specify whether participant food tracking was intended, scaffolded, or avoided (Tables 1, 2). Epilepsy KDT consensus guidance frames tracking intensity as individualized without specifying decision criteria (12, 13). In adjacent literatures, implementation of supported self-management in secondary mental health services depends heavily on organizational conditions including staffing, training, and leadership, not only on patient capacity (108). Reporting standards for complex nonpharmacologic interventions require specification of what participants were expected to do, not only what providers delivered (23, 26).
Assumptions about patient capacity	Rationale for teaching, not teaching, or only partly teaching tracking. Symptom burden, cognitive load, digital literacy, support needs, patient preference, uptake, and actual performance of tracking tasks.	Willingness to attempt tracking, need for prompts or support, refusal, partial uptake, and reasons for non-use or nonteaching.	Distinguish clinician judgment from demonstrated participant performance. Avoid binary framing of can track versus cannot track. Report timing of instruction, level of support, and criteria used to decide who was taught to track or not and at what level of participant involvement.	Observational studies of clinical decision-making, cross-sectional surveys of practitioner criteria, repeated measures designs comparing assumed and demonstrated tracking performance, prospective cohort designs, grounded theory, interpretive phenomenological analysis, and sequential exploratory mixed methods.	Recovery-oriented psychiatric rehabilitation research challenges the assumption that serious mental illness precludes meaningful self-management skill acquisition (38, 58). Recent meta-reviews document that up to three-quarters of psychiatric patients retain decision-making capacity and that impairments are modifiable with intervention (35). Cognitive remediation meta-analyses report that patients with higher baseline symptom severity show larger treatment gains, directly inverting deficit-based capacity assumptions (36). Whether similar capacity exists for food tracking skill acquisition in psychiatric KMT has not been examined.
Psychosocial treatment functions of participant food tracking	Behavioral activation, self-efficacy, successful task completion, treatment ownership, self-direction, confidence in independent dietary execution, graded skill acquisition, and likelihood of continuation after support decreases.	Perceived usefulness, engagement with tracking, willingness to continue, confidence growth over time, and whether support can be reduced without loss of fidelity.	Specify whether tracking is being taught as a skill, a support tool, a self-monitoring practice, or some combination. Report timing, support intensity, and whether participants are expected to grow toward greater independence. Avoid reducing participant food tracking to adherence measurement only.	Repeated measures designs, pragmatic cohort studies, mediation analyses nested within broader KMT trials, experience-based co-design, longitudinal mixed methods designs, and interpretive phenomenological analysis.	Self-management skill acquisition in serious mental illness depends on autonomy-supportive, competence-building delivery that supports self-efficacy and internalized motivation (58, 59). Graded tasks, self-monitoring with feedback, and professional human support are established as effective behavior change techniques across chronic disease interventions (60). Whether participant food tracking in psychiatric KMT carries psychosocial treatment functions including behavioral activation, mastery, and self-efficacy building has not been examined (68).
Psychosocial risks and burdens of participant food tracking	Perceived burden, frustration, cognitive overload, perfectionism, shame, avoidance, dropout, partial uptake, abandonment of tracking, and reasons for nonuse.	Tolerance of tracking demands, distress tolerance associated with tracking, support required to continue, and whether burden changes over time, by setting, or by level of participant involvement (Figure 2).	Report negative psychological effects directly rather than assuming that nonuse or attrition reflects simple nonadherence. Distinguish early learning burden from sustained harmful burden.	Repeated measures designs, ecological momentary assessment, pragmatic cohort studies with prospective burden measurement, mediation analyses nested within broader KMT trials, adverse event monitoring frameworks nested within broader KMT trials, interpretive phenomenological analysis, and convergent mixed methods.	Dietary self-monitoring adherence declines predictably, with fewer than half of participants sustaining tracking beyond 10 weeks in behavioral interventions (56, 57). Adjacent literatures document both burden and psychological risk, including associations between calorie tracking and disordered eating (50, 53) and the experience of diet as a source of both self-efficacy and guilt in adults with serious mental illness (46, 55). Whether participant food tracking in psychiatric KMT carries similar risks, different risks, or net benefit has not been examined.
Function of participant generated tracking data	Whether tracking is used for self-monitoring, clinician review, dietitian adjustment, shared decision making, treatment ownership, or adherence estimation only. Frequency of review and whether dietary modifications follow from the participant food tracking data.	Burden and benefit for participants, burden and utility for clinicians, frequency of review, and perceived value of tracking for diet adjustment or treatment engagement.	Distinguish the presence of tracking from the function of tracking. Avoid assuming that participant generated food records or macronutrient tracking data are used in the same way across studies or programs.	Chart review and audit designs, cross-sectional surveys of clinician and dietitian data review practices, workflow mapping studies, pragmatic cohort studies, ethnographic observation, thematic analysis, and convergent mixed methods.	The function dietary self-monitoring data serve varies across behavioral intervention studies, including whether data are reviewed, by whom, and whether clinical action follows (32, 109). Behavior change technique taxonomies classify self-monitoring and feedback as distinct active ingredients requiring separate specification (110, 111). Reporting standards for complex interventions require that each component be described by its rationale, mechanism, and clinical use (23). What function participant-generated food tracking data serve in psychiatric KMT has not been specified in the published literature.
Continuity after support decreases or stops	Ability and/or motivation to continue KMT after discharge, reduction in coaching, reduction in ketogenic professional contact, or transition from more structured to less structured care or support. Retention, continuation, and changes in how independently the diet is maintained.	Confidence in maintaining the diet, need for ongoing prompts, reasons for discontinuation after support changes.	Report what participants were actually taught to do before support decreased. Distinguish continuation during active support from continuation after support changes.	Prospective longitudinal designs initiated before planned support reduction, repeated measures spanning pre and post support reduction phases, ecological momentary assessment, narrative inquiry, and sequential explanatory mixed methods.	Adherence to ketogenic dietary therapy in epilepsy declines over time, and predictors of continuation versus discontinuation after professional support decreases have not been identified (72). No adaptive stepped-care study in the adult health behavior literature has tested systematic reduction of support for treatment responders (73). Whether participant food tracking skill acquisition during supported psychiatric KMT supports dietary continuation after professional support decreases has not been examined.
Clinical outcomes associated with participant food tracking	Psychiatric symptom outcomes, persistence, treatment fidelity, burden, continuity, and clear description of the tracking approach used.	Whether participant food tracking is sustainable across the duration of KMT treatment. Participant-reported acceptability of tracking demands over time.	Caution in interpreting symptom differences in relation to tracking unless the tracking method, level of participant involvement (Figure 2), support structure, and tracking intensity are described clearly enough to permit comparison.	Comparative cohort studies with standardized delivery reporting, pragmatic trials, secondary analyses of broader KMT studies with strong delivery reporting, systematic reviews and meta-analyses as the literature matures, thematic analysis of patient defined outcomes, and sequential explanatory mixed methods.	The relationship between levels of participant food tracking and clinical outcomes for adults has not been established, with available evidence finding no association between ketone-based adherence proxies and seizure response (112). No validated measure links participant dietary recording to clinical outcomes in adult ketogenic dietary therapy (72). Whether participant food tracking precision affects psychiatric symptom outcomes in KMT remains an open and unstudied question.
Role of support persons in participant food tracking	Support person involvement in initiation, scaffolding, or execution of food tracking on behalf of the participant. Whether involvement was intended as temporary scaffolding toward independence or as a sustained proxy model. Support person role, relationship to participant, and any role preparation.	Participant willingness to involve a support person, support person burden, and whether supported tracking transitions to independent tracking over time.	Distinguish support person assisted tracking from participant independent tracking. Report whether support person involvement was clinically indicated, participant preferred, or a default assumption. Avoid conflating supported tracking with participant self-management.	Prospective longitudinal designs tracking transition from supported to independent tracking, comparative descriptive studies, cross-sectional surveys of practitioner rationale for support person involvement, dyadic qualitative designs, narrative inquiry, and sequential exploratory mixed methods.	The role of support persons in initiating or scaffolding food tracking on behalf of adults with psychiatric illness has not been evaluated in the published psychiatric KMT literature. Adjacent chronic illness and eating disorder literatures establish that caregiver involvement can build competence when autonomy-supportive or create dependency when substitutive, and that staged transition from caregiver-directed to patient-directed management is feasible when intentionally structured (115–118). Whether similar dynamics apply to support person involvement in food tracking for psychiatric KMT remains unknown.
Participant characteristics that may moderate food tracking feasibility and outcomes	Psychiatric diagnosis, symptom severity, cognitive functioning, demographic characteristics, cultural context, health literacy, digital literacy, food security status, and prior experience with dietary self-management.	Whether food tracking feasibility, acceptability, or burden differs by participant characteristics. Whether tracking instruction or tools require adaptation for specific populations.	Report participant characteristics with enough specificity to permit comparison across studies. Avoid treating psychiatric KMT populations as homogeneous. Distinguish whether tracking approaches were adapted to participant characteristics or delivered uniformly.	Moderation analyses nested within broader KMT trials, stratified descriptive studies, cross-sectional surveys of practitioner adaptation practices, community-based participatory research, interpretive phenomenological analysis, and convergent mixed methods.	No published epilepsy KDT or psychiatric KMT study has examined whether participant food tracking feasibility, adherence, or outcomes differ by sociodemographic background, cultural context, health literacy, or food security (13, 72). Epilepsy health equity frameworks document significant disparities across the care continuum but have not been applied to participant food tracking in published research (113, 114). Whether participant food tracking in psychiatric KMT requires adaptation to participant characteristics remains unstudied.

This table outlines research priorities and decision points concerning KMT delivery and participant food tracking in psychiatric ketogenic metabolic therapy. The table is organized around distinct but related priority domains that are interconnected in clinical practice. For each priority domain, it specifies outcomes and variables to report, feasibility and acceptability indicators, design and reporting priorities, candidate study designs, and research rationale with key sources.

Different levels of participant food tracking generate different types of dietary records, and the records may inform clinical decisions about dietary adjustment and treatment response, or may sit unused as documentation of adherence. Whether dietary records shape clinical action in any given delivery model varies across studies and across programs, and the presence of tracking in a study report does not on its own indicate what the data are doing for the participant or the clinician. The level of clinical engagement with participant-generated dietary records is itself a feature of how participant food tracking is delivered, and one that may differ between programs that look otherwise similar in their tracking instruction. Clinical engagement with the records, when it occurs, ranges from real-time review that informs ongoing dietary adjustment to retrospective review that confirms adherence after the period of interest has passed, and the form of engagement may shape what tracking is for in a way that is independent of how the tracking itself was taught.

Programs delivering psychiatric KMT can examine their own criteria for who is taught to track and at what level of participant involvement. Research measuring outcomes in psychiatric KMT may benefit the literature by capturing the functions tracking may serve for the participant in addition to comprehensive evaluations of adherence rates, because tracking evaluated only through adherence measures will appear identical across delivery models that may in fact be producing very different effects on the participant. Studies reporting delivery model details should differentiate professionally managed approaches, participant tracking approaches, and hybrid models that combine or sequence them across phases of treatment to increase visibility of what is being clinically delivered. Programs designing tracking instruction and studies reporting delivery model details should specify whether participant involvement was intended to remain stable across treatment or to change over time, and what criteria were used to make that decision.

The clinical reasoning behind that variation currently sits with individual practitioners and programs and is not consistently published or described. These criteria can be studied as clinical reasoning rather than inferred from the delivery models they produce. Practitioner assumptions and expectations about what level of participant food tracking an individual can manage shape the decision to teach tracking or not, and the field has not distinguished clinician judgment from demonstrated participant performance in the published psychiatric KMT literature. Observational studies of clinical decision-making and surveys of practitioner criteria can make those distinctions visible in ways the current literature does not. Until practitioner decision criteria and clinician engagement with records are visibly reported, the field is studying the output of clinical reasoning it cannot evaluate that may improve patient outcomes.

Dietary continuation after professional support decreases may look different in participants whose food tracking involvement was high during active treatment than in those whose involvement was low, and the reasons participants give for continuing or stopping have not been studied in relation to what they were taught to do with food. Participant characteristics may moderate whether food tracking is feasible for a given individual and what outcomes that tracking produces, and the dimensions that may matter include psychiatric diagnosis, symptom severity, cognitive functioning, cultural context, health literacy, digital literacy, food security, and prior experience with dietary self-management. Support person involvement in the initiation, scaffolding, or execution of food tracking on behalf of the participant may shape that trajectory differently depending on whether the support is reduced over time as the participant takes over, or whether the support person continues tracking in the participant’s place indefinitely as a sustained proxy model.

The implications of this framework are not that psychiatric KMT programs should converge on a single model or level of participant involvement in food tracking, but that decisions currently made need to be reported clearly enough to support research that can inform future practice. Comparative questions about participant food tracking cannot be answered unless studies specify what participants were taught to do and what the goals or purpose participant-generated food records served within the study or program. The field also needs visibility into how decisions about tracking were made, and whether participants successfully continued KMT after professional support decreased or ended. Continuation of participant food tracking during active support and continuation after support changes are different timepoints and provide different data that are potentially relevant to outcomes. Without adequate description, two studies both reporting participant food tracking may in fact be describing different participant tasks and different ways the records participants produced were used clinically.

## 8 Discussion

The level of participant involvement in food tracking encouraged or expected of adults receiving psychiatric KMT is a clinical decision the field has not yet examined directly. The three unresolved clinical questions are not intended to be read as a causal sequence. They identify different uncertainties surrounding that decision, including the capacity assumptions that shape who is encouraged to track and at what level, the possibility that tracking may function as burden or as treatment work, and the relation between short-term professional management and the degree of treatment independence a participant may later need to maintain. The continuum in Figure 2 describes variation in level of participant involvement of food tracking as part of their dietary implementation, and the three questions identify why placement on that continuum may matter clinically and what remains unexamined about the decisions that place participants there. The operational definition of participant food tracking provided in this framework is consistent with this structure because it narrows the construct under examination to participant-generated dietary recording without treating all points on the continuum as instances of tracking. The distinction between estimated and weighed tracking may also be useful to clinicians and researchers as differences in tracking precision change what the participant is asked to do, the kind of record produced, and what those records can support in clinical adjustment.

Several established theoretical traditions help locate this framework within existing clinical and behavioral work. Self-efficacy theory is relevant to the possibility that repeated completion of food tracking tasks may shape perceived capability, treatment ownership, and confidence in independent dietary execution (63). Behavioral activation is relevant to the possibility that tracking may function as treatment work through the daily completion of a structured task rather than through the data it produces (67, 68). The literature on behavior change distinguishing initiation from maintenance is relevant to the distinction drawn here between active support and continuation after support changes (78). Recovery-oriented psychiatric rehabilitation is relevant because it centers participant agency, skill acquisition, and the development of self-management capacity rather than assuming those capacities are absent (38, 58, 59). Future empirical work could also draw on broader implementation science frameworks, including the Consolidated Framework for Implementation Research (83), RE-AIM (84), and Normalization Process Theory (85, 86), to examine how organizational context, reach, adoption, workflow integration, implementation fidelity, maintenance, and routine embedding shape psychiatric KMT delivery.

The framework developed in this conceptual analysis does not claim that participant food tracking is therapeutic in itself or that greater participant involvement in food tracking is preferable to professionally managed dietary implementation. It does not claim that clinician assumptions about patient capacity are uniformly wrong. The framework holds each of these as an open question the field can investigate, which is what makes them research priorities rather than conclusions. It is developed for clinical consideration and research with adults receiving clinician-supervised psychiatric KMT across the range of settings and diagnoses represented in the published literature reviewed in this analysis. It does not address pediatric populations or acute presentations in which participants cannot engage with food and does not extend to self-directed ketogenic treatment without clinical oversight. It assumes that psychiatric KMT is a legitimate clinician-supervised treatment for the diagnoses it addresses and that dietary precision may play a relevant role for therapeutic ketosis in adults. The adjacent literatures on self-management and behavior change are treated as relevant enough to inform hypothesis generation but not as direct evidence for psychiatric KMT. The operational definitions of participant food tracking used in this analysis are proposed as workable for the field to test and refine, not as settled terms.

Decisions about whether and how a participant receiving psychiatric KMT is taught to track their food are being made every day in the field of metabolic psychiatry, without a shared framework for examining the reasoning behind those decisions or a shared language for describing what the tracking is doing when it occurs. The consequences of those decisions land on the participant inside these delivery models in ways the field has not yet characterized. To the participant exploring KMT as a potential treatment for serious mental illness, these decisions are deeply personal. The framework developed in this conceptual analysis aspires to give clinicians, researchers, and programs a shared way to ask what participant food tracking in psychiatric KMT is doing for the people receiving it, and to pursue the answers these questions deserve.

## Supplementary material

SUPPLEMENTARY TABLE 1 Narrative descriptions of KMT delivery and participant food tracking as reported in each included publication.

SUPPLEMENTARY TABLE 2 Estimated and weighed participant food tracking approaches and the reporting specificity each supports.
