# Supplementary material for: Participant food tracking in psychiatric ketogenic metabolic therapy: an unresolved clinical, psychosocial, and implementation question
Source: Front Nutr. 2026 Aug 6;13:1879746. doi: 10.3389/fnut.2026.1879746 (PMC13489925; doi:10.3389/fnut.2026.1879746)
Supplement: Supplementary file 1 [file Table_1.DOCX]

**Supplementary Table 1. Narrative descriptions of reported delivery model and participant food tracking in the published psychiatric KMT literature**

| **Clinical studies and program evaluations** | |
| --- | --- |
| **Study/design** | **Delivery model and participant food tracking as reported** |
| Pacheo et al., 1965 Pilot study | A ketogenic diet was added to ongoing inpatient treatment for ten women with chronic schizophrenia in the earliest published psychiatric application of a ketogenic dietary intervention (Pacheco et al., 1965). The publication documents diet implementation as part of inpatient care but does not describe diet supervision, meal provision, or participant involvement in dietary monitoring (Pacheco et al., 1965). |
| Calabrese et al., 2022  Open label pilot study | A therapeutic ketogenic diet for weight-restored adults with anorexia nervosa was introduced through a two-day immersion program followed by four to eight weeks of home maintenance of nutritional ketosis before ketamine infusions (Calabrese et al., 2022). During immersion, study staff, participants, and support persons prepared and shared meals in an informal group setting. After returning home, participants received daily monitoring calls reduced to biweekly by the end of four weeks, with weekly optional group meetings for additional support. The paper does not specify whether participant food tracking was taught or used (Calabrese et al., 2022). |
| Danan et al., 2022  Retrospective analysis of clinical care | A professionally managed inpatient ketogenic diet intervention for adults with severe, persistent mental illness, including bipolar disorder, schizoaffective disorder, and major depressive disorder, provided three dietitian-prepared meals per day, one daily snack box, and a list of approved foods (Danan et al., 2022). The treating psychiatrist met individually with each participant six days per week to monitor clinical progress and provide dietary education and support. Dietary adherence was estimated using physician interviews, nursing observations, and participants' daily food journals. The publication describes daily food journals as part of adherence estimation but does not specify the journal format or the level of detail participants were expected to record (Danan et al., 2022). |
| Needham et al., 2023  Feasibility pilot study | A six- to eight-week outpatient feasibility trial of a modified ketogenic diet in euthymic individuals with bipolar disorder provided individualized dietary prescriptions specifying daily calories and macronutrient grams divided into portions, with recipes adapted for each participant and weekly remote dietitian contact for adherence support (Needham et al., 2023). Participants completed a three-day food diary before diet initiation to facilitate personalization and planning. The study incorporated a behavioral adherence component informed by the COM-B framework (Michie et al., 2011). During the intervention, dietary prescription changes were made by the dietitian based on ketone levels, weight changes, and participant preferences, but participant food tracking procedures during the intervention period were not described (Needham et al., 2023). |
| Rigby et al., 2025  Mixed-methods process evaluation process of Needham et al., 2023 | A mixed-methods process evaluation of the same cohort found that participants were actively calculating fat and carbohydrate ratios during meal preparation, supported by individualized dietitian guidance including tailored recipes and weekly diet review meetings (Rigby et al., 2025). One participant described the cognitive demands of these calculations as considerable, particularly in the context of medication-related cognitive effects. The process evaluation documented that participants managed dietary composition through ongoing collaboration with the dietitian rather than through a formalized food recording protocol, and participant food logging was not part of the study procedures (Rigby et al., 2025). |
| Sethi et al., 2024  Pilot trial | A four-month supported outpatient ketogenic intervention for adults with schizophrenia or bipolar disorder with existing metabolic abnormalities provided a one-hour teaching session, educational handouts at each visit, ketogenic cookbooks, recipes, a resource list, and a personal coach for the duration of the study (Sethi et al., 2024). Participants completed regular visits with the study physician and coaches checked in weekly to discuss food-related questions. Participants were instructed to reduce and monitor carbohydrate intake to approximately 20 g per day. The paper does not describe formal food logging procedures or structured daily recording of dietary intake (Sethi et al., 2024). |
| Decker et al., 2025; pilot study | A partially provisioned outpatient ketogenic intervention for college students with major depressive disorder was delivered over 10 to 12 weeks with extensive dietetic support (Decker et al., 2025). Participants received a dietary education session before baseline, personalized education and ongoing contact with the dietetic team through a messaging application, and ten prepackaged ketogenic meals at baseline as a visual guide for meal composition. After the initial provision, participants selected and prepared foods at home with additional provision of selected shelf-stable ketogenic food items and electrolyte products. The paper does not specify whether participant food tracking was taught or used (Decker et al., 2025). |
| Bellamy, 2026 retrospective evaluation | A retrospective evaluation of a fully remote online group KMT program for adults with varied psychiatric diagnoses described a group-based supported self-management model delivered over six months by a practitioner trained in KMT implementation (Bellamy, 2026). The intervention included personalized macronutrient targets, live education, weekly support sessions, and a moderated private online community. Participants prepared all of their own meals. The paper states that participants were asked to track macronutrient intake on a food app, described as a learning tool (Bellamy, 2026). |
| Brietzke et al., 2026  open-label pilot feasibility trial | An open-label pilot feasibility trial of a medically supervised ketogenic diet for adults with moderate to severe major depressive disorder or bipolar depression consisted of a two-week dietitian-supervised induction phase followed by a twelve-week maintenance phase with weekly dietitian consultations delivered virtually or in person (Brietzke et al., 2026). Participant food tracking was described in part. Participants maintained daily food records and monitored macronutrients, and these were reviewed by the dietitian at weekly consultations. The specific method or format of the daily food records was not reported (Brietzke et al., 2026). |
| Gao et al., 2026  RCT | A randomized clinical trial of a remotely delivered outpatient ketogenic intervention for adults with treatment-resistant depression provided three prepared ketogenic meals per day, snacks, written materials, and weekly support sessions of up to 30 minutes with a dietitian or trained coach for six weeks. Participants who preferred to prepare their own meals received weekly food vouchers instead. The paper does not specify whether participant food tracking was taught or used (Gao et al., 2026). |
| Liang et al., 2026  single-arm pilot study | In a 12-week single-arm outpatient pilot study of adults with binge-eating disorder, a structured ketogenic diet was delivered by a registered dietitian trained in ketogenic therapy, with two initial educational sessions, weekly then bi-weekly support calls, and personalized meal plans, recipes, and grocery lists provided. The paper describes daily food records but does not specify the format or the level of detail participants were expected to record (Liang et al., 2026). |
| Frank et al., 2026  single-arm feasibility trial | A single-arm feasibility trial of adults with anorexia nervosa who were mildly underweight or weight-restored described a 14-week outpatient ketogenic therapy intervention delivered with study dietitian support (Frank et al., 2026). Participants received ketogenic meals through a meal delivery service or prepared their own meals under dietitian guidance, with weekly psychiatrist and dietitian meetings. Participant food tracking was described in part with participants submitting post-meal photographs for dietitian review. The report does not describe a formal daily food record or app-based macronutrient logging (Frank et al., 2026). |
| **Case reports and case series** | |
| **Study/design** | **Delivery model and participant food tracking as reported** |
| Yaroslavsky et al., 2002  Letter to the editor, single case report | Ketogenic dietary treatment was implemented in a 49-year-old woman with severe treatment-resistant rapid-cycling bipolar disorder, with agreement from the patient, her family, and multidisciplinary staff (Yaroslavsky et al., 2002). The intervention began with a 48-hour fast, followed by a classic ketogenic diet for two weeks and then replacement of dietary fats with MCT oil during a one-month trial. The report does not identify a dietary professional involved in the intervention and does not specify whether participant food tracking was taught or used (Yaroslavsky et al., 2002). |
| Phelps et al., 2013  case series | Two outpatient case studies described prolonged ketogenic diets maintained for two and three years in women with type II bipolar disorder. The report included examples of foods consumed and one participant's estimated macronutrient distribution but does not specify who designed or supervised the diet or whether a dietary professional was involved. No participant food tracking information was reported (Phelps et al., 2013). |
| Palmer, 2017  Letter to the editor, two case studies | Two case studies of schizoaffective disorder described ketogenic diets adopted in outpatient settings. The report included foods consumed in each case but did not indicate whether the diet was designed or supervised by a dietary professional or whether any educational or support structure was provided. The report does not specify whether participant food tracking was taught or used (Palmer, 2017). |
| Palmer et al., 2019  Two case studies | Two adult women with longstanding schizophrenia independently initiated and maintained ketogenic diets in outpatient settings over periods of five and twelve years (Palmer et al., 2019). The publication does not describe dietary education, professional dietary supervision, or how the dietary intervention was structured or supported. Whether participant food tracking was taught or used is not addressed in the report (Palmer et al., 2019). |
| Carmen et al., 2020  case series | A case series described three adults with obesity who reported binge eating and food addiction symptoms and self-managed ketogenic diets in outpatient settings with professional support from obesity medicine physicians (Carmen et al., 2020). Patients received macronutrient guidelines with carbohydrates restricted to 20 to 30 g per day, dietary education, food lists, and regular follow-up visits. Patients were asked to track daily carbohydrate intake and completed dietary recall questionnaires at follow-up visits. The specific method or format of carbohydrate tracking was not described in the publication (Carmen et al., 2020). |
| Norwitz et al., 2023  case series | A retrospective case series of three adults with severe, treatment-refractory anorexia nervosa described self-adopted high-fat animal-based ketogenic diets identified through telemedicine interviews and chart review (Norwitz et al., 2023). Diet adoption was prompted by online educational materials, social media recommendations, and internet research, and one patient was later followed by a nutritionist specializing in ketogenic diets. The report did not describe a structured clinical delivery model, meal provision, or consistent professional supervision at diet initiation. One patient reported mentally noting approximate caloric intake to maintain weight, but participant-generated recording of food intake was not described (Norwitz et al., 2023). |
| Laurent, 2024  single case report | A retrospective case study of an adult with bipolar II disorder and treatment-resistant depressive symptoms described KMT delivered remotely through twice-weekly virtual support sessions over a 21-week period (Laurent, 2024). The intervention included individualized macronutrient targets and dietary education, with carbohydrate consumption reduced over two weeks to a target of 30 g total carbohydrate per day. The professional role of the person providing dietary support was not reported. Participant food tracking was reported as app-based macronutrient monitoring with dietary data reviewed during support sessions (Laurent, 2024). |
| Calabrese et al., 2024  case series | A retrospective case series of three adults with major depression, generalized anxiety, and comorbid psychiatric conditions described personalized whole-food animal-based KMT delivered in a specialized metabolic psychiatry outpatient practice (Calabrese et al., 2024). Participants received twice-weekly visits with an experienced ketogenic registered dietitian who designed and supervised the dietary intervention. Participant food tracking was reported as daily photo journaling of food intake (Calabrese et al., 2024). |
| Bellamy and Laurent, 2025 retrospective case report | A retrospective case report of an adult with PTSD, ADHD, binge-eating disorder, and multiple comorbid psychiatric diagnoses described KMT delivered through an eight-week psychoeducation program with ongoing professional and peer support (Bellamy & Laurent, 2025). The intervention was delivered by a practitioner trained in KMT implementation, with twice-weekly support calls and a private community platform. Participant food tracking was reported as app-based macronutrient tracking, used for approximately one month and then discontinued as meals became consistent (Bellamy & Laurent, 2025). |
| Laurent et al., 2025  retrospective case study | A retrospective case study of an adult with chronic treatment-resistant major depressive disorder described KMT delivered remotely through an online education and support program that included self-paced education, live group sessions, and peer support (Laurent et al., 2025). The diet was initiated and supervised by a ketogenic-informed nutrition professional within a treatment team that included a medical doctor and a psychotherapist. Participant food tracking was reported as app-based macronutrient logging (Laurent et al., 2025). |
| Calabrese, 2025  case report | A case report of an adult with obsessive-compulsive disorder and ulcerative colitis described KMT delivered in a specialized outpatient metabolic psychiatry clinic over 12 weeks (Calabrese, 2025). The diet was personalized and developed by a ketogenic registered dietitian, with twice-weekly dietitian visits for one month followed by weekly visits for the remainder of treatment. The participant implemented KMT at home without food delivery services. Participant food tracking was reported as photo journaling of food intake (Calabrese, 2025). |
| Laurent et al., 2025  retrospective case series | A retrospective case series of two individuals with schizoaffective disorder described KMT delivered in outpatient settings with medical oversight (Laurent et al., 2025). In both cases, the diet was initiated by a ketogenic-trained professional within a treatment team that included a prescriber and a psychotherapist, with KMT support provided through twice-weekly virtual sessions. Participant food tracking was reported as app-based macronutrient tracking in both cases (Laurent et al., 2025). |
| MacDonald and Palmer, 2025 case series | A case series of three adults with obsessive-compulsive disorder described self-initiated and self-managed ketogenic diets maintained in outpatient settings without professional dietary supervision (MacDonald & Palmer, 2025). The publication describes foods consumed in each case but does not describe dietary education or a structured KMT delivery model. The report does not specify whether participant food tracking was taught or used (MacDonald & Palmer, 2025). |
| Laurent and Tague, 2025  retrospective case report | A retrospective case report of an adult with treatment-resistant obsessive-compulsive disorder described a modified ketogenic diet that was self-initiated and implemented at home without professional dietary guidance (Laurent & Tague, 2025). The patient independently determined macronutrient targets and maintained the diet without structured dietary supervision over a period extending beyond the initial treatment phase. Participant food tracking was reported as daily recording of dietary intake using a personal spreadsheet (Laurent & Tague, 2025). |
| Murray et al., 2025  case report | A case report of an adult with treatment-resistant schizophrenia and metabolic syndrome described a five-week medical ketogenic diet delivered in an inpatient hospital setting (Murray et al., 2025). Meals were planned by the hospital dietary department, delivered to the patient's room, and monitored by hospital staff on the unit. The report described staff monitoring of meal consumption rather than participant-generated recording of food intake (Murray et al., 2025). |
| Newiss, 2025  case report | A retrospective case report of an adult with schizophrenia described a self-initiated carnivore ketogenic diet maintained across multiple care settings including a rehabilitation hospital and later in the community (Newiss, 2025). After discharge, the subject consulted a nutritional therapy practitioner who provided dietary guidance for approximately two months, after which the subject self-managed the diet without practitioner support. The report describes self-prepared meals and foods consumed but does not describe participant-generated recording of food intake (Newiss, 2025). |
| Laurent, 2026  retrospective case report | A retrospective case report of an adult with treatment-resistant post-traumatic stress disorder described KMT delivered remotely through virtual sessions with a KMT professional over 25 weeks (Laurent, 2026). Professional support included an initial one-hour consultation followed by sessions at weekly to biweekly intervals that transitioned to monthly over the course of treatment. The report described the prescribed dietary pattern and foods consumed but did not describe participant-generated recording of food intake (Laurent, 2026). |
| Rioux et al., 2026  case report | A case report of an adult with bipolar II disorder and comorbid personality disorder and alcohol use disorder described a 3-month modified ketogenic diet implemented at home in outpatient psychiatric care (Rioux et al., 2026). The report described ketogenic diet education before initiation, psychiatric follow-up during the intervention, prescribed daily capillary ketone monitoring, and no dietitian follow-up. The report described the prescribed dietary pattern but did not describe meal provision, foods consumed, or participant-generated recording of food intake (Rioux et al., 2026). |

Supplementary Table 1 Description. Each entry in this supplementary table is a narrative description of one publication in the psychiatric KMT literature. Narratives describe what the publication stated about how KMT was delivered and what the publication stated about whether participants were taught to track their dietary intake. Inferences beyond what the publication stated were not made. The provided narratives provide the basis for the categorical assignments in Tables 1 and 2.
