# Supplementary material for: Participant food tracking in psychiatric ketogenic metabolic therapy: an unresolved clinical, psychosocial, and implementation question
Source: Front Nutr. 2026 Aug 6;13:1879746. doi: 10.3389/fnut.2026.1879746 (PMC13489925; doi:10.3389/fnut.2026.1879746)
Supplement: Supplementary file 2 [file Table_2.DOCX]

**Supplementary Table 2. Descriptive distinction between estimated and weighed participant food tracking in psychiatric KMT**

| Tracking modality | Description | Example in psychiatric KMT | Reporting implication |
| --- | --- | --- | --- |
| Estimated food tracking | Participant records intake using household measures, food labels, visual estimates, generic database entries, photographs, or portion estimates rather than food-scale weights. | A participant enters foods into an app as “2 eggs,” “1 cup spinach,” or “1 tablespoon butter,” tracks daily carbohydrate grams from labels, or submits meal photos for review without weighing foods. | Reporting may include what participants were asked to record, whether portions were estimated, what tool was used, and whether records were reviewed by a clinician or dietitian. |
| Weighed food tracking | Participant records intake after measuring foods or ingredients on a food scale before logging. | A participant weighs ingredients in grams before entering them into an app or spreadsheet to meet daily or per-meal macronutrient targets. | Reporting may include whether foods were weighed, whether targets were daily or per meal, what training was provided, and whether weighed records informed dietary adjustment. |

**Supplementary Table 2 Description.** This table contrasts estimated and weighed participant food tracking in psychiatric KMT. Examples are illustrative and are intended to clarify the participant task and reporting details associated with each modality. The table is descriptive rather than prescriptive.
